# Trajectory of Food Insecurity and Its Association with Longitudinal Mental Health and Sleep Outcomes in Adolescents from Economically Disadvantaged Families

**DOI:** 10.3390/nu13051696

**Published:** 2021-05-17

**Authors:** Ting-Hsuan Lee, Jen-Hao Kuo, Chia-Yi Liu, Yi-Fang Yu, Carol Strong, Chung-Ying Lin, Chih-Ting Lee, Meng-Che Tsai

**Affiliations:** 1 School of Medicine, College of Medicine, National Cheng Kung University, Tainan 70101, Taiwan; asdsky33lee@gmail.com (T.-H.L.); thomas42913@gmail.com (J.-H.K.); i54066518@mail.ncku.edu.tw (C.-Y.L.); 2 Department of Public Health, National Cheng Kung University Hospital, College of Medicine, National Cheng Kung University, Tainan 70101, Taiwan; azurestone705@gmail.com (Y.-F.Y.); carol.chiajung@gmail.com (C.S.); 3 Institute of Allied Health Sciences, College of Medicine, National Cheng Kung University, Tainan 70101, Taiwan; cylin36933@gmail.com; 4 Department of Family Medicine, National Cheng Kung University Hospital, College of Medicine, National Cheng Kung University, Tainan 70101, Taiwan; christina.unicorn@gmail.com; 5 Department of Pediatrics, National Cheng Kung University Hospital, College of Medicine, National Cheng Kung University, Tainan 70101, Taiwan

**Keywords:** adolescent, food insecurity, mental health, sleep outcome, trajectory analysis

## Abstract

Background: Adolescence is a critical transition period in the course of human development. Although food insecurity (FI) has been shown to be associated with adverse mental health and sleep outcomes in US adolescents, there is a paucity of research examining the relationships between FI, mental health, and sleep outcomes in Taiwanese adolescents. Furthermore, it is unknown how the change of FI over time (i.e., the trajectory of FI) is related to health outcomes. Methods: The data come from the Taiwan Database of Children and Youth in Poverty, which is a national longitudinal project measuring FI in five survey waves (2009–2017). We employed group-based trajectory modeling to classify various FI trends over the five waves using STATA. Furthermore, a generalized estimating equation analysis was conducted with FI trajectories as the independent variable to see how FI trajectory is related to mental health and sleep outcomes. Results: In total, 1921 participants aged 12–18 years in the first wave were deemed valid for the analysis. We classified the participants into four FI trajectory groups: persistently low FI (24.8%), persistently moderate FI (64.7%), declining from high to low FI (4.1%), and food-secure groups (6.4%). As compared to food-secure adolescents, the persistently moderate FI group was more likely to have mental problems (β = 0.30, [95% confidence interval 0.21–0.38]), while the other FI groups were only marginally associated with mental health problems. Moreover, adolescents in the persistently low FI group (β = 0.13, [0.02–0.23]) and persistently moderate FI group (β = 0.39, [0.29–0.48]) were found to have more sleep problems than those in the food-secure group. Conclusions: Our study describes the FI profile of adolescents from economically disadvantaged families and the difficulties they might encounter. With this information, healthcare providers can aid adolescents in the early stages of mental health problems and provide guidance when appropriate.

## 1. Introduction

Food insecurity (FI) is typically defined as the uncertain ability or the inability to procure food or enough food, making affected individuals unable to live a healthy life or feel unsatisfied [1]. Despite the increase in national wealth and food production in Taiwan, 7.8% of the overall population has been reported as being underfed or regularly hungry, according to a recent national survey [2]. In growing children, FI may jeopardize one’s health and development. Prior research has shown that food-insecure children are more likely to have a poorer health status and experience colds, stomachaches, and headaches more frequently than their food-secure counterparts [3]. Moreover, children’s behavioral, emotional, and academic outcomes are also affected by FI [4]. For instance, it has been shown that 6–11-year-old food-insecure children have significantly lower arithmetic scores and are more likely to have repeated a grade, seen a psychologist, and had difficulty getting along with other children [5]. In adults, FI is associated with functional limitations and chronic diseases [6]. A systematic review and meta-analysis have also revealed that depression, anxiety, and sleep disorders are more prevalent in households with FI among a mixed population, including college freshmen, seniors, veterans, HIV+ individuals, low-income caretakers, and the general population [7]. A subgroup analysis suggests that the association between FI and depression is consistent across specific populations, although gender difference may exist [6]. The present study primarily focuses on the issue of FI during the stage of adolescence, where FI may pose more threats to normative development during this critical period of physical and psychological transition between childhood and adulthood. Food-insecure individuals may experience stress, anxiety, and shame [8], and they have a higher likelihood for obesity [9], which can manifest as an elevated risk for poor mental health and sleep problems. Several studies have indicated that an association between FI and a wide range of mental disorders in adolescents exists [10,11,12]. For example, a strong association was found between an increase in FI and the odds of past-year mental disorder, particularly in families with a low household income and high relative deprivation [10]. The association may have had a dosage effect, given that as the severity of household FI increased, so did the odds of mental disorder [13].

Previous research generally involved a cross-sectional design and operated FI using a single item or a set of items relevant to FI in the analysis [10,13,14]. This may restrict the issue of FI to a fixed status that does not take its time-varying trait into account when analyzing the impacts. In fact, the status of food security is likely to change over time. How the change of FI over time (i.e., the trajectory of FI) can impact adolescents’ psychosocial development has not been fully characterized. We hypothesize that persistent FI is associated with poorer mental health and sleep disturbances in adolescents. Specifically, we aim to investigate the association between trajectories of FI, mental health, and sleep outcomes among Taiwanese adolescents from economically disadvantaged families.

## 2. Methods

### 2.1. Study Population

A subset of data on participants aged 12–18 years in wave 1 (in 2009) was obtained from the Taiwan Database of Children and Youth in Poverty (TDCYP), which is comprised of a nationally representative cohort of children and youths from families receiving governmental subsidies or social services provided by the Taiwan Fund for Children and Families [15,16]. The survey took place biennially on the same subsidized families, until the recipients of the subsidies or services were disqualified. The subjects (children, teenagers, and their families) were randomly sampled based on stratification by administrative municipalities in Taiwan. Those who have reading difficulties, or mental or cognitive disabilities were excluded from recruitment. Eligible participants were accessed, and they and their parents or guardians gave informed consent to the study when returning the questionnaire. A total of five waves (wave 1 in 2009, wave 2 in 2011, wave 3 in 2013, wave 4 in 2015, and wave 5 in 2017) of data collection were used for this analysis. The study was approved by the Institutional Review Board of the National Cheng Kung University Hospital.

### 2.2. Measures

#### 2.2.1. Predictor Variable

Referencing the original Core Food Security Module developed by the US Department of Agriculture, and considering the availability of data in the TDCYP, Wang et al. [17] constructed and validated a similar measure on food security, consisting of dichotomous items on food, modified eating behaviors, concerns about food availability, and hunger levels within the past year [18]. Specifically, the measure had a total of four items: (1) eating less than three meals and no other snacks in a day, (2) starving due to a lack of money, (3) skipping breakfast or lunch in order to save money, and (4) financial difficulties in paying for lunch. To align with prior research, we added up all the item scores to create a four-item FI scale (FIS) [19]. Concerning the missing data, we divided the FIS scores by the number of answered questions, if more than two thirds of the questions were answered at any given wave. Thus, the FIS was rescaled from 0 to 1. This scale indicates the degrees of FI, with a higher score representing higher FI.

#### 2.2.2. Outcome Variables

In this study, the outcome variables are mental health and sleep problems. Mental health was measured using the Taiwan version of the short 5-item Brief Symptom Rating Scale (BSRS-5). A Likert scale from 1 (*none*) to 5 (*extremely*) was applied to rate each item. A sum score was used to reflect psychological wellbeing. Given some missing data, we divided the sum score by the number of answered questions, if more than two thirds of the questions were answered at any given wave. Sleep problems were assessed by a single item retrieved from BSRS-5, indicating whether subjects had sleep problems or difficulty falling asleep. Outcome variables were only assessed in waves 3–5.

### 2.3. Covariates

We included gender (male vs. female), family structure (living with two parents vs. other family structures), parental citizenship (foreign vs. domestic parents), and monetary household incomes per person in the analyses. These covariates were all assessed in wave 1 [18].

### 2.4. Analytic Strategy

To model the trajectory of FI, which was assumed to be heterogeneous in growth functions, we employed group-based trajectory modeling (GBTM) to classify various trends of FIS over the entire period of adolescence using STATA. Subjects with more than two missing assessments in FIS were excluded to ensure that the trajectory analysis was based on a minimum of three data points. Missing FIS values were imputed with sample means for the rest of the subjects who remained in the final analysis. First, we identified food-secure individuals who scored 0 on FIS across multiple waves and treated them as a reference group in following regression analyses. Additionally, complete cases with non-missing data in all waves were entered into the GBTM analysis to determine the optimal number of class memberships and the shape of growth curves. We tested the shape of the trajectory groups by including higher-order polynomial growth factors (linear, quadratic, and cubic time factors). We used the Bayesian information criterion (BIC) to determine the optimal number of trajectory groups, and the average posterior probability measure and the weighted odds of correct classification to examine the best-fit trajectory shape [20]. Once the shape and the number of the best solution were determined, all food-insecure individuals were entered into the GBTM, and each was assigned to one of the latent classes using the modal class assignment procedure and the posterior distribution information pertaining to GBTM. Lastly, we examined the association between the trajectory pattern of food security and psychological and sleep outcomes using a generalized estimating equation (GEE) analysis, as the outcome variables were repeatedly measured in waves 3 to 5. With respect to the food-secure adolescents, we calculated the coefficient (β) and 95% confidence intervals (CIs) of depression symptomatology and sleep problems in the other trajectory groups in our GEE model, after controlling for gender, family structure, and monthly family income per person. Except for the GBTM, all the other analyses were performed using SPSS 25.0 (SPSS Inc., Chicago, IL, USA).

## 3. Results

### 3.1. Demographic Characteristics

The final sample included a total of 1921 adolescents with a mean age of 14.4 (±1.9) years, with 56.3% being female (Table 1). Among the participants, 1798 (93.6%) experienced some levels of FI at some point during the study period. Moreover, only 21% of this cohort lived with both parents (missing data = 5, 0.3%), and 5.4% had at least one parent of foreign nationality (missing data = 6, 0.3%). Nearly 40% of individuals lived with a household income of less than 3000 New Taiwan Dollars per person-month.

### 3.2. Food Insecurity Trajectories

**Figure 1:**
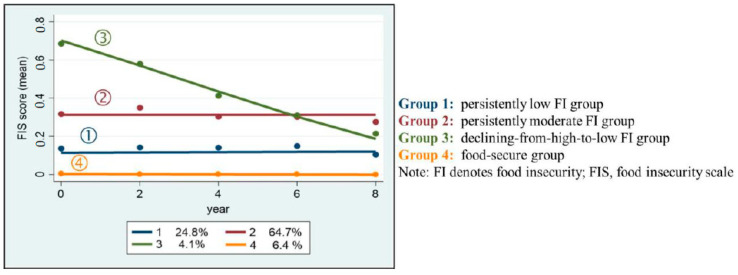
Food insecurity trajectories over time.

Based on five waves of FIS scores (missing data = 538, 28% for one wave and 415, 21.6% for two waves), the three-class solution for the data on food-insecure individuals was the best fit in GBTM because of its exceptionally low BIC. Accordingly, the following four groups were created: persistently low FI, persistently moderate FI, declining from high to low FI, and no FI (food-secure individuals) (Figure 1). The persistently low FI group (*n* = 477, 24.8%) was estimated to have an intercept at 0.1 on the FIS in wave 1 and retained the same value across all waves, while the persistently moderate FI group (*n* = 1242, 64.7%) had an intercept at 0.3 and remained stable. The last group, with FI declining from high to low (*n* = 79, 4.1%), had an estimated intercept at 0.7 on the FIS in wave 1 and dropped to 0.2 in wave 5. There were other individuals who did not report any form of FI across all waves, and were thus classified under the food-secure group (*n* = 123, 6.4%).

### 3.3. Mental Health and Food Insecurity Trajectory

The GEE analysis found that compared to the food-secure group, individuals with persistently moderate FI were more likely to have mental health problems (β = 0.30, 95% CI = 0.21–0.38), while the other trajectory groups were only marginally associated with mental health problems. Moreover, mental health problems differed between genders in that females were more likely to have mental problems than males (β = 0.11, 95% CI = 0.06–0.16). Otherwise, there was no significant association between FI and the family structure or household income (Table 2).

### 3.4. Sleep Outcomes and Food Insecurity Trajectory Membership

Adolescents with persistently low FI (β = 0.13, 95% CI = 0.02–0.23) and persistently moderate FI (β = 0.39, 95% CI = 0.29–0.48) were found to have more sleep problems than their food-secure counterparts (Table 3). There was a dosage effect in the association between levels of FI and sleep problems. Moreover, females were more likely to have sleep problems than males (β = 0.13, 95% CI = 0.07–0.19).

## 4. Discussion

To the best of our knowledge, this present study is the first to demonstrate a link between FI trajectories and mental health and sleep outcomes in economically disadvantaged youth. Using the GBTM, we classified the participants into four trajectory groups based on their food security status. Only 6.4% of the participants were food-secure, while the rest experienced some amount of FI at some time point during their adolescence. As compared to their food-secure peers, individuals across different FI trajectories had a higher likelihood of experiencing mental health and sleep problems.

Aligned with prior research conducted in the United States [10], FI in our cohort was also associated with poor mental health outcomes. In our analysis, we observed a gradient effect on the association between FI and mental health problems. Compared to the food-secure group, there was a 30% increase in the likelihood of mental health problems in the persistently moderate food-insecure group, while this likelihood had a 9% increase in the persistently low FI group. The difference in likelihood remained statistically significant after making adjustments for socioeconomic measurements, such as household incomes, suggesting that FI is a strong and independent predictor of adolescent mental health outcomes. Although a prior study found a correlation between FI and household material hardships [17], our finding may indicate that FI is a more pernicious form of material deprivation that leads to more psychological adversity among food-insecure individuals than those living simply in low-income families without FI.

In addition, our analysis also found a similar pattern of association between FI and sleep problems. A prior study of a population-based adult sample in the United States found that women with very low food security reported lower sleep durations than women who were fully food-secure [21]. Contrarily, men who experienced any form of FI had longer sleep latencies than fully food-secure men [21]. A biological model involving the hypothalamic–pituitary–adrenal axis has been proposed to understand the process of how FI perturbs physical functions [22,23]. In a study on monkeys, food-deprived female monkeys manifested marked individual cortisol responses and compromised normative maternal–infant rearing behaviors [22]. In a human study, FI in early life was significantly associated with a flattened cortisol slope among young children, indicating a disturbed diurnality of cortisol excursion. Taken together, FI can lead to chronic stress that may perturb the homeostatic function of the hypothalamic–pituitary–adrenal axis, further leading to sleep disturbances.

Our findings further extend the current body of knowledge by showing differential associations between FI trajectories over time and mental health outcomes. At baseline, the declining from high to low food-insecure group was estimated to have the highest level of food security, which declined gradually over time as compared to the three other food-insecure groups. Strikingly, the association analysis found the risk of adverse mental health and sleep outcomes to be attenuated for this group as compared to those in the persistently moderate food-insecure group. That being said, the negative effects of FI could be reduced if levels of FI declined over time. This finding provides supporting evidence of a pressing need to design interventions that will stabilize or reverse FI during adolescence. As FI still prevails among economically disadvantaged populations despite the presence of governmental subsidization, increasing their access to nutritious foods through the establishment of food supply stations linked with convenience stores or restaurants, or by enhancing health literacy in the importance of healthy eating among adolescents, may be advocated when addressing psychological health issues in a population living in poverty [19]. Worthy of attention is the fact that although we adopted a longitudinal approach for data analysis, the results should be understood as correlational. Group membership that was mainly based on the data-driven analysis was thus not necessarily a cause of the development of mental health and sleep problems. Potentially, the causal relationship might be bidirectional, in that FI increases the risk of poor emotional health, and poor emotional health increases the risk of FI [24]. Individuals with a mental illness, such as mood and substance disorders, were more likely to report persistently high FI, as indicated in a survey on homeless adults [25]. Further research is needed to investigate whether a similar relationship can be found in child and adolescent populations.

This study has some limitations. First, except for gender, household income, and family structure, other potential confounding factors were not controlled in our study. For example, parental education was not included because of missing data: 67% and 33% were missing on father’s and mother’s education, respectively. Second, the survey on sleep outcome was only a single question retrieved from BSRS-5. Participants scored their sleep outcome from 1 (*none*) to 5 (*extremely*) based on a composite item assessing whether they had difficulty falling asleep, waking up early, or experienced disturbed sleep quality. This may not be able to objectively capture sleep latencies and sleep durations and might thus cause biased results. Third, the Taiwan Fund for Children and Families provides subsidies based on household units. Hence, adolescents who leave their original family, including those who are homeless, escaping from family, or joining a gang, might have been left out of the survey sample. These juvenile subjects, after leaving home, are unable to receive financial support and may account for a significant proportion of those potentially affected by FI. Given the lack of data on this unreached population, the findings from our study may not be applicable to their situations. Finally, in light of a small number of individuals assigned to the reference group, the association analysis may be underpowered to detect statistical significance, particularly for the declining from high to low FI group, which is also a small sample size. Further research with a larger participation is needed to verify our findings.

## 5. Conclusions

According to the present study, levels of FI were associated with mental health and sleep outcomes in a dose-dependent manner among economically disadvantaged youth. Amelioration in FI, corresponding to a downward curve on FIS over time, was linked to a lower degree of mental health and sleep problems. These findings may have clinical and public health implications in that support for food security can be leveraged to improve mental health and sleep outcomes in this vulnerable group, since mental health issues and FI are often seen as intertwined chronic conditions. Public health policy makers or governmental subsidy sectors should consider fund allocation and food delivery via reliable venues to ensure food security. From the clinical perspective, healthcare providers are also advised to assess food security in poverty-affected youth and to provide appropriate guidance on nutrition when addressing their mental health and sleep issues. 

## Figures and Tables

**Table 1:** Demographic information of participants (*n* = 1921).

Characteristic	*n*	%
Food insecurity group		
Declining from high to low FI group	79	4.1%
Persistently moderate FI group	1242	64.7%
Persistently low FI group	477	24.8%
Food-secure group	123	6.4%
Age, years, mean (SD)	14.4	(± 1.9)
Gender		
Female	1081	56.3%
Male	840	43.7%
Family structure		
Parents of foreign nationalities	104	5.4%
Living with two parents	403	21%
Household incomes per person-month * (NTD)		
≤3000	770	40.1%
3001–6000	792	41.2%
≥6001	359	18.7%

FI denotes food-insecure; SD, standard deviation; NTD, New Taiwan Dollar. * The median of household incomes per person-month was approximately 18,000 NTD in 2009.

**Table 2:** The association between mental health problems and food insecurity trajectory.

Variables	β	95% CI		*p* Value
		Lower	Upper	
Food insecurity group				
Declining from high to low	0.187	−0.023	0.398	0.081
Persistently moderate	0.297	0.212	0.382	<0.001
Persistently low	0.091	−0.001	0.182	0.052
Gender				
Female	0.113	0.063	0.163	<0.001
Household incomes (per person-10,000 NTD)	0.013	−0.085	0.110	0.798
Family structure				
Parents of foreign nationality	−0.099	−0.201	0.004	0.059
Living with two parents	0.041	−0.019	0.101	0.182

CI denotes confidence interval; NTD, New Taiwan Dollar.

**Table 3:** The association between sleep problems and food insecurity trajectory.

Variables	β	95% CI		*p* Value
		Lower	Upper	
Food insecurity group				
Declining from high to low	0.223	−0.012	0.458	0.063
Persistently moderate	0.387	0.290	0.484	<0.001
Persistently low	0.128	0.023	0.234	0.017
Gender				
Female	0.127	0.065	0.188	<0.001
Household incomes (per person-10,000 NTD)	0.050	−0.117	0.126	0.936
Family structure				
Parents of foreign nationality	−0.124	−0.253	0.005	0.059
Living with two parents	0.013	−0.064	0.089	0.747

CI denotes confidence interval; NTD, New Taiwan Dollar.

## Data Availability

The TDCYP dataset is managed and archived by the Taiwan Fund for Children and Families. Although it is free and open to the public, accessing the dataset requires registration. All the waves could be found in the following link: https://tfcfrg.ccf.org.tw/tpoor/main.htm?pid=33&ID=1 (accessed on 1 July 2020).
